# Axial spondyloarthritis patients with comorbid fibromyalgia feel worse, work less and more often try multiple biological therapies: results from a population-based, cross-sectional study investigating the discriminative capacity of pressure algometry

**DOI:** 10.1007/s00296-026-06140-1

**Published:** 2026-05-23

**Authors:** Annie Brink, Elisabeth Mogard, Elisabet Lindqvist, Jonas Sagard, Carmen Roseman, Mats Geijer, Jack Kvistgaard Olsen, Johan K. Wallman, Tor Olofsson

**Affiliations:** 1 Faculty of Medicine, Department of Clinical Sciences Lund, Rheumatology, Lund University, Kioskgatan 5, Lund, 221 85 Sweden; 2 Department of Rheumatology, Skåne University Hospital, Lund, Sweden; 3 Department of Radiology, Sahlgrenska University Hospital, Region Västra Götaland Gothenburg, Sweden; 4 Department of Radiology, Institute of Clinical Sciences, University of Gothenburg, Sahlgrenska Academy, Gothenburg, Sweden; 5 Department of Clinical Sciences Lund, Diagnostic Radiology, Lund University, Lund, Sweden; 6 The Parker Institute, Bispebjerg and Frederiksberg Hospital, Copenhagen, Denmark

**Keywords:** Fibromyalgia, Spondyloarthritis, Ankylosing spondylitis, Algometry

## Abstract

To estimate prevalence and associated factors of comorbid fibromyalgia in axial spondyloarthritis (axSpA), and to explore discriminative capacity (for discerning fibromyalgia) of algometry-assessed pain sensitivity measures. AxSpA patients from the population-based SPARTAKUS cohort evaluated for fibromyalgia (1990 ACR criteria) were included (*n* = 243; r-axSpA/nr-axSpA = 165/78). Factors associated with fibromyalgia were cross-sectionally analyzed by logistic/linear regression, and discriminative potential of algometry-assessed measures estimated by ROC-curve analysis. A fibromyalgia prevalence of 9% was demonstrated, with a higher frequency in female than male patients (17%/2%;adjusted *p* < 0.001) and more than a doubling in frequency per 5-unit BMI increase (adjusted *p* < 0.002). Fibromyalgia was associated with several disease/work outcomes, including higher disease activity, lower quality-of-life, and less employment although without association to swollen joints/CRP. Additionally, patients with (versus without) fibromyalgia had higher bDMARD exposure (19%/5% had tried ≥ 3 bDMARDs; adjusted *p* = 0.009), and were more often on opioids (62%/16%;adjusted *p* < 0.001). Algometry-assessed measures displayed a sensitivity/specificity of 79%/70% (pain threshold) and 71%/83% (pain tolerance) for fibromyalgia differentiation (positive/negative predictive values 16%/99% and 23%/98%, respectively). Fibromyalgia is a frequent comorbidity in axSpA, more common in female patients/patients with higher BMI, and associated with worse levels of patient-reported disease/work outcomes. Our findings highlight the challenge of assessing axSpA disease activity when fibromyalgia is present and suggest a role for algometry as a complementary, evaluator-independent assessment tool, potentially useful for fibromyalgia rule-out but less so for rule-in. The results further suggest that fibromyalgia may be associated with DMARD overtreatment, reflecting a need for earlier and more precise targeting.

**Supplementary Information:**

The online version contains supplementary material available at 10.1007/s00296-026-06140-1.

## Introduction

Inflammation control has become an attainable target in most axial spondyloarthritis (axSpA) patients owing to an increasing number of biological/targeted synthetic disease-modifying anti-rheumatic drugs (b/tsDMARDs). Despite this, many patients still suffer from refractory, generalized pain, disconnected from inflammation; often in its most severe form fibromyalgia (FM). The prevalence of FM has been estimated to 2–3% in the general population [1, 2], higher in women [1], whereas meta-analyses have demonstrated a FM prevalence ranging up to 30% in axSpA [3, 4]. Most previous findings are however limited to patients with radiographic axSpA (r-axSpA; formerly referred to as ankylosing spondylitis [AS]), while less is reported for non-radiographic axSpA (nr-axSpA). Earlier studies of FM encompassing both axSpA subtypes are often performed in rather small, consecutively recruited cohorts or restricted to patients with a certain therapeutic regime [5–8]. Hence, data from unrestricted, population-based cohorts, comprising both nr-axSpA and r-axSpA, are largely lacking.

A main feature of FM is central pain sensitization [9], but the etiology of FM remains largely unknown [10]. Risk factors of FM in the general population include high BMI, low physical activity, smoking, long-lasting pain, and stress [11, 12]. Moreover, emerging data suggest a role for immunologic abnormalities, such as altered cytokine/chemokine profiles [13] and antibodies towards satellite glial cells [14]. Despite the high FM prevalence observed in axSpA, risk factors of its occurrence have been scantly studied.

FM is often accompanied by fatigue, irritable bowel syndrome and psychiatric comorbidity [10]. Together with widespread pain, these features could potentially entail worse patient-reported outcomes in axSpA with concomitant FM [15]. Some previous axSpA studies have demonstrated higher disease activity [7, 16], more work impairment and lower quality-of-life [7], when FM was present. It has also been shown that axSpA patients with FM are more likely to receive bDMARD therapy [7], and have a lower retention rate on their first tumor necrosis factor (TNF) inhibitor [16]. This has raised concerns that FM-induced worsening of axSpA disease activity scores could lead to more (and potentially improper) DMARD treatment, where further data from well-characterized cohorts, encompassing both axSpA subtypes, would be valuable.

Discerning which axSpA patients suffer from comorbid FM is hence of great importance, albeit challenging since differentiating between inflammatory pain from axial involvement/enthesitis and non-inflammatory generalized pain can be difficult. Consequently, more reliable tools for pain assessment/monitoring would be valuable. Computerized cuff pressure algometry (CPA) is a technique to more objectively measure muscle/deep tissue pain sensitivity [17], and significantly lower pain threshold/tolerance have been displayed in primary FM compared to healthy controls using this method [18]. Whether CPA could distinguish between FM/no-FM in axSpA is so far not known.

Based on the above and by using a population-based and thoroughly classified axSpA cohort, including both r-axSpA and nr-axSpA, we aimed to: (1) estimate the prevalence of comorbid FM; (2) explore factors associated with FM; and (3) examine if CPA-assessed pain sensitivity measures could differentiate between FM and no-FM.

## Materials and methods

### Setting and patient population

SPARTAKUS is a population-based, clinical cohort of established axSpA from southern Sweden [19]. All patients from a defined geographical area of Skåne county with ≥ 1 visit at the Rheumatology Department, Skåne University Hospital, between 2011 and 2014, and an ICD-10 diagnosis suggestive of axSpA, were identified. Patients with M45.9, M46.0 and M46.1 (indicating axial involvement) were directly invited to enroll, whereas patients with undifferentiated SpA diagnoses (M46.8, M46.9) had to report back pain ≥ 3 months before age 45 to be eligible. Enrolled patients underwent a predefined study protocol encompassing questionnaires (lifestyle, disease activity, physical function, current/previous treatments and quality-of-life); clinical examinations by a rheumatologist and physiotherapist; blood sampling (HLA-B27, C-reactive protein [CRP]); FM assessment (1990 ACR criteria) [20], and pain sensitivity examination by CPA [21]. X-ray/magnetic resonance imaging (MRI) of the sacroiliac joints was performed via a thorough algorithm [19], as needed for axSpA classification (by ASAS [Assessment of SpondyloArthritis international Society]/modified New York criteria) [22, 23]. In total, 266 patients fulfilled axSpA criteria (r-axSpA = 180; nr-axSpA = 86). Twenty-two had missing data for ≥ 1 item needed for FM classification and one patient was excluded due to antibiotics-treated febrile infection at the study visit, yielding 243 patients for the current cross-sectional study (Supplementary Figure S1). Ethical approval was granted by the Lund Regional Ethical Review Board (Dnr. 2015/436; approval date 2015-08-20). All patients gave written informed consent in compliance with the Declaration of Helsinki, adhered to throughout the project.

### Fibromyalgia assessment

Fibromyalgia (FM) was assessed by the ACR 1990 classification criteria to provide a conservative, well-established capture mechanism, including a thorough tender point examination of all patients by the experienced study rheumatologists [20].

### Lifestyle factors

Smoking was categorized as ever/never. Alcohol use/physical activity were categorized according to guidelines from the Swedish National Board of Health and Welfare (Socialstyrelsen) (see Supplementary Methods)[24].

### Disease and health measures

Swollen/tender joint counts (of 66/68) and enthesitis (Maastricht Ankylosing Spondylitis Enthesitis Score [MASES]) were clinically evaluated, whereas pain, fatigue, and global health were assessed by Visual Analogue Scales (VAS; 0–100 mm). AxSpA disease activity was assessed by Ankylosing Spondylitis Disease Activity Score using CRP (ASDAS-CRP) [25] and Bath Ankylosing Spondylitis Disease Activity Index (BASDAI) [26]; functional limitations by Bath Ankylosing Spondylitis Functional Index (BASFI) [27]; spinal mobility by Bath Ankylosing Spondylitis Metrology Index (BASMI) [28]; quality-of-life by EuroQol 5-Dimensions (EQ-5D) utility [29], and aerobic capacity via Åstrand’s submaximal cycle ergometer test [30].

### Pharmacological treatments

Treatments (from questionnaires/medical records) included ongoing/previous conventional synthetic DMARDs (csDMARDs) and bDMARDs, as well as ongoing NSAIDs, analgesics (paracetamol, opioids), and other pain-influencing medications (tricyclic anti-depressants, selective serotonin/serotonin-norepinephrine reuptake inhibitors [SSRIs/SNRIs].

### Work and activity measures

Work/activity limitations were assessed by the Work Productivity and Activity Impairment Questionnaire: General Health (WPAI: GH) [31]: including current employment, absenteeism, presenteeism, overall work impairment and overall activity impairment (Supplementary Methods).

### Pain sensitivity by pressure algometry

Pain sensitivity measures (pain threshold, pain tolerance, temporal summation of pain) were assessed with computerized cuff pressure algometry (CPA) [17, 21]. Temporal summation of pain reflects patient-reported pain intensity over a 10-minute sequence with constant, individually adapted pressure [32], generating a temporal summation index (TSI) (Supplementary Methods).

### Statistics

Demographics, axSpA phenotype, and lifestyle factors − hypothesized as potential risk factors of FM − were analyzed by logistic regression with FM (yes/no) as dependent variable, and univariately significant factors introduced into a multivariate model. Variables rather hypothesized as being impacted by FM, were analyzed with FM (yes/no) as independent variable using linear/logistic regression, univariately and age/sex-adjusted (if ≥ 10 events in the smallest group of a studied outcome). 95% confidence intervals (CIs) were obtained via non-parametric, bias-corrected accelerated bootstrapping for skewed continuous outcomes (normality-tested by Shapiro-Wilks test). ROC-curve analysis was adopted to explore discriminative capacity of CPA-assessed pain sensitivity measures (for distinguishing FM from no-FM). Bootstrap-based 95% CIs were calculated for AUC estimates to account for the limited/imbalanced sample size. The Youden index was used to determine cut-off points. Due to limited sample size, no train/test split was applied and all analyses were performed on the whole study sample. For details, see Supplementary Methods. The Benjamini-Hochberg (BH) procedure was adopted to account for multiple comparisons. Methodology adhered to the STROBE standard of the EQUATOR Network (related checklist provided as supplementary file).

## Results

The study cohort (*n* = 243; r-axSpA/nr-axSpA 165/78) had a mean age/symptom duration of 51/25 years; 53% being male and 86% HLA-B27 positive (Table 1). Disease activity was low and 42% had ongoing bDMARD treatment. Excluded patients did not differ from the study cohort in demographic/disease parameters (Supplementary Table S1).

**Table 1:** Characteristics of the axSpA study population

	Total (*n* = 243)	r-axSpA (*n* = 165)	nr-axSpA (*n* = 78)
Age, years	51 (13)	53 (13)	47 (12)
Male sex, n (%)	130 (53%)	100 (61%)	30 (38%)
Symptom duration, years	25 (14)	28 (14)	20 (11)
BMI, kg/m2	26 (5)	26 (4)	26 (5)
HLA-B27 positivity, n (%)	209 (86%)	139 (85%)	70 (90%)
SJC (of 66)	0.2 (0.7)	0.2 (0.8)	0.2 (0.4)
TJC (of 68)	3.9 (6.1)	3.5 (5.5)	4.7 (7.1)
CRP, mg/L	3.4 (4.6)	3.8 (5.2)	2.5 (2.9)
ASDAS-CRP	1.8 (0.9)	1.8 (0.9)	1.8 (0.9)
BASDAI	3.0 (2.2)	3.0 (2.2)	3.0 (2.1)
NSAIDs, ongoing, n (%)	154 (63%)	104 (63%)	50 (64%)
bDMARDs, ongoing, n (%)	103 (42%)	73 (44%)	30 (39%)
csDMARDs, ongoing, n (%)	48 (20%)	32 (19%)	16 (21%)
Fibromyalgia, n (%)	21 (8.6%)	15 (9.1%)	6 (7.7%)

All results are presented as mean (SD) unless otherwise indicated

Missing data: symptom duration 1, HLA-B27 positivity 1, SJC 5, TJC 5, CRP 1, ASDAS-CRP 6, BASDAI 7, NSAIDs 1.

*ASDAS-CRP* ankylosing spondylitis disease activity score using CRP, *AxSpA*, axial spondyloarthritis, *BASDAI* Bath ankylosing spondylitis disease activity index, *bDMARDs* biologic disease-modifying anti-rheumatic drugs, *BMI* body mass index; csDMARDs, conventional synthetic DMARDs, *CRP* C-reactive protein; *HLA-B27* human leukocyte antigen B27, *NSAIDs* non-steroid anti-inflammatory drugs, *r-axSpA* radiographic axSpA, *SJC* swollen joint count, *TJC* tender joint count

### Fibromyalgia prevalence and association to background factors

**Fig. 1:**
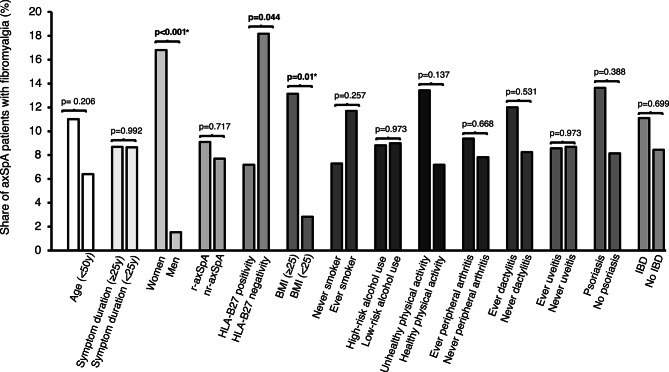
Frequencies of fibromyalgia in axSpA patients, stratified according to different background characteristics, axSpA phenotypes and lifestyle factors; with p-values from comparisons between strata of each variable by univariate logistic regression. Asterisks (*) indicate significant association (p-value < 0.05) in multivariate analysis, including factors that were significant in the univariate analyses. High-risk alcohol use: weekly consumption of > 14 standard units or > 5 units/one occasion (men), and > 9 standard units or > 4 units/one occasion (women). Unhealthy physical activity: <150 min weekly exercise of at least moderate intensity. Cut-offs for alcohol use/physical activity according to recommendations from the Swedish National Board of Health and Welfare. Missing data: symptom duration 1, HLA-B27 1, smoking 14; alcohol use 9, physical activity 9. AxSpA, axial spondyloarthritis; BMI, body mass index; HLA-B27, human leukocyte antigen B27; IBD, inflammatory bowel disease; nr-axSpA, non-radiographic axSpA; r-axSpA, radiographic axSpA; y, years

**Fig. 2:**
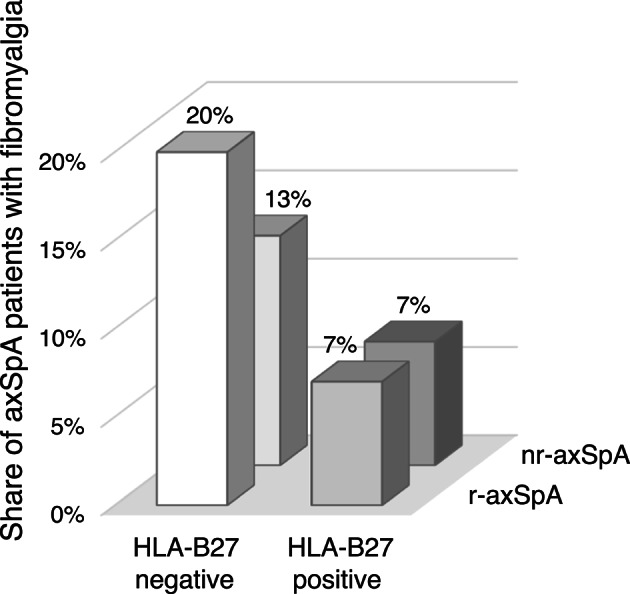
Frequencies of fibromyalgia (FM) in axSpA patients (*n* = 243), stratified according to axSpA subtype and HLA-B27 status. Numbers above boxes indicate the frequency of FM in each group. Total number of patients (irrespective of FM) in each group: HLA-B27 positive r-axSpA, 139; HLA-B27 negative r-axSpA, 25; HLA-B27 positive nr-axSpA, 70, HLA-B27 negative nr-axSpA, 8. Missing data: HLA-B27 status 1. AxSpA, axial spondyloarthritis; nr-axSpA, non-radiographic axSpA; r-axSpA, radiographic axSpA

The prevalence of comorbid fibromyalgia (FM) was estimated at 8.6%, with a higher percentage in female than male patients: 16.8% versus 1.5% (OR 12.9 [95%CI 2.9–56.9), *p* < 0.001) (Fig. 1; Supplementary Table S2). BMI was significantly associated with presence of FM (OR 2.02 [95%CI 1.29–3.17] per 5-unit increase, *p* = 0.01) which was also the case for HLA-B27 status with FM in 18%/8% of HLA-B27 negative/positive patients (OR 2.87 [95%CI 1.03–8.04], *p* = 0.04). In multivariate analysis, significance remained for sex (adjusted OR 13.4 [95%CI 3.0–61.1, *p* < 0.001) and BMI (adjusted OR 2.17 [95%CI 1.34–3.51], *p* = 0.002), unaltered after the BH procedure. No association to FM was seen for age, symptom duration, axSpA subtype, smoking or unhealthy physical activity. When specifically exploring FM prevalence in relation to axSpA subtype and HLA-B27 status, the highest estimate was seen in HLA-B27 negative r-axSpA patients (20%) (Fig. 2).

### Association between comorbid fibromyalgia and various outcomes measures

#### Disease and health outcomes

In both univariate and age/sex-adjusted analyses, presence of FM was associated with worse axSpA disease activity, with an adjusted mean difference in ASDAS-CRP of 0.8 (95%CI 0.4 to 1.1; *p* < 0.001) for FM versus no-FM (Table 2). Patients with FM reported worse levels of pain, fatigue and global health, more functional limitations, and lower quality-of-life. All significances remained after the BH procedure. No significant associations were displayed between presence of FM and more objective measures of inflammation/structural alterations (CRP, swollen joints, physiotherapist-assessed spinal mobility or aerobic capacity).

**Table 2:** Associations between comorbid fibromyalgia and various disease/health measures in axSpA

	Mean (SD)		Univariate		Age- and sex adjusted	
	FM (n = 21)	No FM (n = 222)	ß (95% CI)	*P* value	ß (95% CI)	*P* value
SJC (of 66)	0.15 (0.67)	0.19 (0.67)	-0.04 (-0.3 to 0.3)	0.792	-0.05 (-0.4 to 0.3)	0.741
TJC (of 68)	16 (6)	3 (5)	13 (10 to 16)	*< 0.001*	11 (9 to 15)	*< 0.001* ^†^
MASES (0–15)	9.9 (2.5)	3.9 (3.4)	6.0 (4.8 to 7.1)	*< 0.001*	4.6 (3.3 to 5.9)	*< 0.001* ^†^
CRP (mg/L)	3.8 (4.5)	3.3 (4.6)	-0.4 (-1.8 to 1.1)	0.637	-0.1 (-1.1 to 1.2)	0.934
ASDAS-CRP	2.6 (0.7)	1.7 (0.9)	0.9 (0.6 to 1.3)	*< 0.001*	0.8 (0.4 to 1.1)	*< 0.001* ^†^
BASDAI (0–10)	5.1 (2.1)	2.8 (2.1)	2.3 (1.3 to 3.3)	*< 0.001*	1.9 (0.9 to 2.8)	*0.002* ^†^
VAS global (0-100; mm)	55 (21)	29 (25)	26 (16 to 35)	*< 0.001*	22 (12 to 33)	*< 0.001* ^†^
VAS pain (0-100; mm)	55 (25)	29 (25)	25 (14 to 35)	*< 0.001*	21 (10 to 32)	*0.002* ^†^
VAS fatigue (0-100; mm)	62 (24)	32 (27)	30 (19 to 40)	*< 0.001*	25 (12 to 33)	*< 0.001* ^†^
EQ-5D utility score*	0.39 (0.33)	0.75 (0.21)	-0.36 (-0.50 to -0.21)	*< 0.001*	-0.34 (-0.45 to -0.23)	*< 0.001* ^†^
BASFI (0–10)	4.2 (2.5)	1.8 (2.0)	2.3 (1.2 to 3.5)	*< 0.001*	2.1 (1.0 to 3.2)	*< 0.001* ^†^
BASMI (0–10)	3.0 (1.4)	3.0 (1.6)	0.04 (-0.7 to 0.8)	0.908	0.3 (-0.3 to 0.9)	0.401
Aerobic capacity (VO2 max)	32 (5)	35 (10)	-2.9 (-9.7 to 4.0)	0.409	-2.2 (-8.4 to 4.1)	0.495

Mean differences (ß) with 95% confidence intervals (CI) from linear regression models, unadjusted and after adjustment for age and sex. Åstrand is a submaximal bicycle ergometer test to estimate aerobic capacity (VO2max)

Missing data in patients with FM: SJC 1, TJC 1, BASDAI 1, EQ-5D 1, BASFI 1, BASMI 1, Åstrand 12. Missing data in the no FM group: SJC 4, TJC 4, CRP 1, ASDAS-CRP 6, BASDAI 6, VAS global 4, VAS pain 4, VAS fatigue 4, EQ-5D 7, BASFI 7, BASMI 7, aerobic capacity 62.

*ASDAS-CRP* ankylosing spondylitis disease activity score using CRP, *axSpA* axial spondyloarthritis, *BASDAI* Bath ankylosing spondylitis disease activity index, *BASFI* Bath ankylosing spondylitis functional Index, *BASMI* Bath ankylosing spondylitis metrology index, *CRP* C-reactive protein, *FM* fibromyalgia; *MASES* Maastricht ankylosing spondylitis enthesitis score, *SJC* swollen joint count; TJC, tender joint count, *VAS* visual analogue scale

† Significant also after applying the Benjamini-Hochberg procedure

*EQ-5D assessed using the standard British time trade-off-based preference set (a utility score of 1 equals full health and 0 equals death)

### Pharmacological treatments

**Fig. 3:**
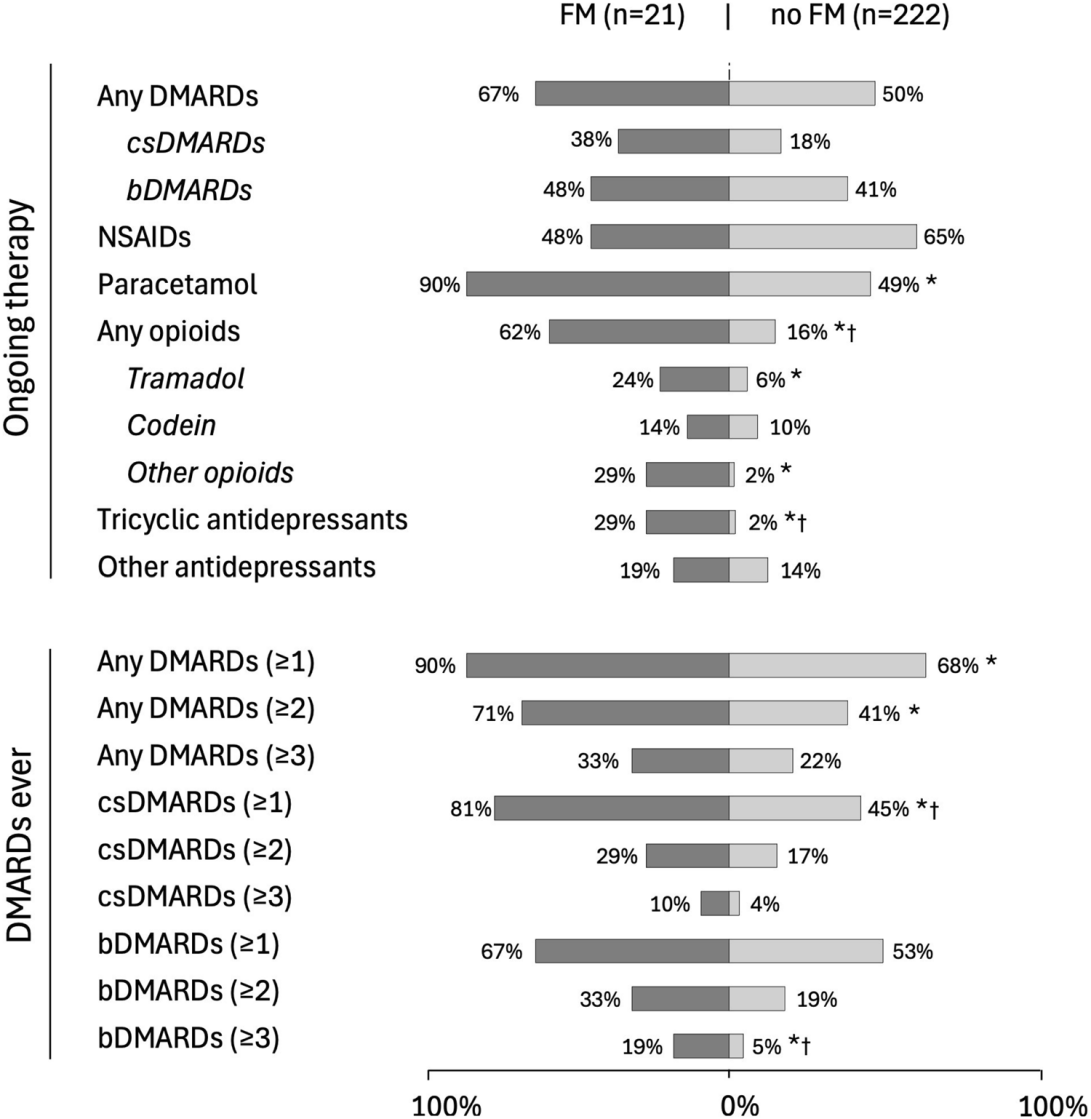
Bar charts display proportions of axial spondyloarthritis (axSpA) patients with ongoing treatments (upper section) and DMARDs ever (lower section), with versus without comorbid fibromyalgia (FM). Asterisks (*) indicate significant association (p-value < 0.05) in age and sex-adjusted logistic regression between comorbid FM and ongoing therapies as well as number of DMARDs ever. (†) indicates significance also after applying the Benjamini-Hochberg procedure. Other antidepressants include selective serotonin reuptake inhibitors (SSRIs) and serotonin-norepinephrine reuptake inhibitors (SNRIs). Missing data in the no FM group: paracetamol 1, any opioids 3, other opioids 3. No missing data in the FM group. DMARDs, disease-modifying anti-rheumatic drugs; bDMARDs, biologic DMARDs; csDMARDs, conventional synthetic DMARDs; NSAIDs, non-steroid anti-inflammatory drugs

Both univariately and in age/sex-adjusted analysis, axSpA patients with comorbid FM had more often received ≥ 1 DMARD (ever) than patients without FM (90%/68%; age/sex-adjusted *p* = 0.032), ≥ 2 DMARDs (71%/41%; adjusted *p* = 0.016) and ≥ 1 csDMARD (81%/45%, adjusted *p* = 0.004). Additionally, 19% with comorbid FM had tried ≥ 3 bDMARDs compared to 5% in the no-FM-group (adjusted *p* = 0.009) (Fig. 3, **Supplementary Table **S3). The FM-group was also more often on ongoing opioids than patients without FM (62%/16%; adjusted *p* < 0.001), paracetamol (90%/49%; adjusted *p* = 0.019), and tricyclic antidepressants (29%/2%; adjusted *p* < 0.001). After the BH procedure, ≥ 1 csDMARD, ≥ 3 bDMARDs, ongoing opioids and tricyclic anti-depressants retained significance, while ≥ 2 DMARDs (*p* = 0.053), ≥ 1 DMARDs (*p* = 0.071), ongoing paracetamol (*p* = 0.054) and tramadol (*p* = 0.071) lost it.

### Work and activity outcomes

Working-age axSpA patients with FM reported a substantially lower employment rate: 48% versus 91% in the no-FM-group (*p* < 0.001 in age/sex-adjusted analysis) (Supplementary Table S4). For employed patients, no significant differences were observed between FM/no-FM regarding absenteeism, presenteeism, or overall work impairment. Still, when comparing overall activity impairment (age-independent and not confined to work tasks), the FM-group reported significantly worse scores (mean difference 28; *p* = 0.002 in age/sex-adjusted analysis [scale 0–100, with 100 representing total activity impairment]).

### Association to algometry-assessed pain sensitivity measures

**Fig. 4:**
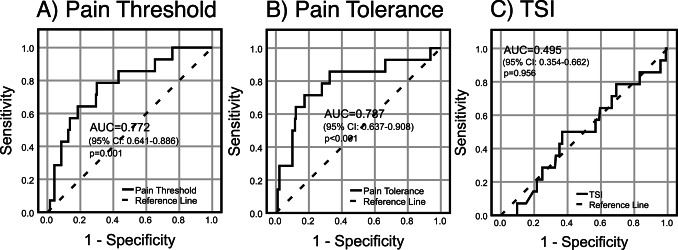
Results from ROC analyses of algometry-assessed pain sensitivity measures: **A** pain threshold, **B** pain tolerance, and **C** temporal summation index (TSI) in relation to presence of comorbid fibromyalgia (FM) in axial spondyloarthritis patients. 95% confidence intervals (CI) for AUC estimates were generated by bootstrapping. Missing data (FM/no FM): pain threshold, 7/32; pain tolerance, 7/32; TSI, 7/37. AUC, area-under-the-curve; ROC, receiver operating characteristics

A lower pain threshold was significantly associated with presence of FM (age/sex-adjusted OR of having FM was 2.08 per 10-unit decrease; *p* = 0.042), as was lower pain tolerance (age/sex-adjusted OR 1.42 per 10-unit decrease; *p* = 0.046) (Supplementary Table S5). No association was displayed between TSI and presence of FM. Regarding discriminative capacity, ROC-curve analyses with bootstrapped-generated confidence intervals revealed an area-under-the-curve of 0.772 (95%CI 0.641–0.886), *p* = 0.001 for pain threshold; 0.787 (95%CI 0.637–0.908), *p* < 0.001 for pain tolerance; and 0.495 (95%CI 0.354–0.662), *p* = 0.956 for TSI **(**Fig. 4**)**. The proposed cut-off point using the Youden index was 24.5 kPa for pain threshold (sensitivity/specificity: 79%/70%), and 42.9 kPa for pain tolerance (sensitivity/specificity: 71%/83%). No cut-off point was determined for TSI due to non-significance. The accuracy/positive predictive value/negative predictive value at the cut-off points were 71%/16%/99% for pain threshold, and 82%/23%/98% for pain tolerance. The corresponding positive/negative likelihood ratios (LR+/LR-) were 2.6/0.3 for pain threshold and 4.1/0.3 for pain tolerance.

### Sensitivity analyses

When regression models for estimating associations of FM with various outcomes were adjusted for sex and BMI (instead of sex and age), no statistical significances were altered, but effect sizes decreased slightly for some variables. Since pain perception can be influenced by other conditions than FM and certain treatments, the association analyses regarding pain threshold/pain tolerance and presence of FM were adjusted for other potentially pain-influencing comorbidities (diabetes, thyroid diseases, peripheral neuropathies, vitamin B12 deficiency, depression and anxiety disorders), ongoing anti-depressants and opioids, yielding similar estimates and no alterations in significances (Supplementary Table S5). For details on comorbidity retrieval (ICD-codes via Region Skånes Vårddatabas [RSVD]), see Supplementary Methods.

## Discussion

### Main results

In the current study from a population-based cohort of thoroughly classified and well-characterized axSpA patients, including both r-axSpA and nr-axSpA, we found an FM prevalence (1990 ACR criteria) of 9%, with a female to male ratio of 11:1. Presence of FM was associated to overweight with roughly a doubling in FM frequency for every 5-unit BMI increase, whereas no significant associations were seen for other proposed risk factors such as age, axSpA subtype, symptom duration, smoking or unhealthy physical activity. We found associations between comorbid FM and several disease/health outcomes in axSpA, with significantly worse scores of practically all instruments fully or partly assessed by self-report: higher disease activity scores; worse levels of pain, fatigue and physical function; and lower quality-of-life. Conversely, no associations were displayed between FM and more objective measures of inflammation/structural alterations such as swollen joints, CRP or spinal mobility. Moreover, only half of the working-age patients with FM were employed compared to 90% without FM. The FM-group was also more likely to have received multiple bDMARDs. Additionally, algometry-assessed pain threshold and pain tolerance displayed decent sensitivity/specificity for differentiation between FM/no-FM in axSpA, with good negative but poorer positive predictive values.

### Previous research

The FM prevalence of 9% demonstrated here is substantially higher than most general population estimates (~ 2–3%) [1, 2], but in the lower spectrum of previously reported frequencies in axSpA [4]. Most previous axSpA studies are, however, limited to r-axSpA and have often been performed in rather small and non-population-based cohorts, yielding varying estimates [3, 4]. Only a few earlier reports have included both r-axSpA and nr-axSpA patients in the analyses. Macfarlane et al. [7] found a FM prevalence of 21% in British axSpA patients, but with FM assessed via the 2011 ACR classification criteria [33], demonstrated to generate elevated prevalence estimates [4, 34]. Baraliakos et al. [5] displayed a FM prevalence of 14% (1990 ACR criteria), although with exclusion of patients who had ever received a bDMARD, whereas Moltó et al. [6] found a prevalence of 16% (1990 ACR criteria), but with analyses restricted to axSpA patients initiating bDMARD therapy. Such approaches, selecting only subsets of axSpA patients and/or usage of different FM criteria, could explain varying prevalence estimates when compared to the present study.

The higher prevalence of FM in female compared to male axSpA patients (17% versus 2%) in the current study, is consistent with previous data from the general population, where a large Scottish study displayed a female/male ratio of 14:1 [35]. Although less pronounced than in our study, results from a British axSpA population by Macfarlane et al. [7] demonstrated a female/male gradient for FM going in the same direction (26% versus 18%), and similar numerical trends have been observed in a few smaller studies [8, 36]. The less marked female/male ratios seen in these studies could at least partly be due to usage of the less conservative 2011 criteria, shown to attenuate FM sex differences as compared to 1990 criteria [35].

Further, the association between higher BMI and presence of FM shown here, is congruent with previous results in axSpA [7] and align with data from the general population [11], highlighting BMI as a potentially modifiable factor with high relevance for FM. Furthermore, in contrast to earlier general population findings [37], smoking and unhealthy physical activity were not associated with presence of comorbid FM in the current study, possibly signaling that these factors might have less relevance for occurrence of comorbid FM in axSpA than for FM in the general population.

We also found a univariate association between HLA-B27 negativity and comorbid FM, consistent with findings from the afore-mentioned study by Macfarlane et al. [7]; results that rebut earlier concerns that primary FM patients with HLA-B27 positivity might incorrectly be classified as nr-axSpA. In fact, the FM prevalence displayed here was highest among HLA-B27 negative patients with r-axSpA (20%)(Fig. 2), resonating well with results from Baraliakos et al. [5], which showed that AS patients fulfilled FM criteria more often than patients with nr-axSpA and that patients with primary FM only fulfilled axSpA criteria (ASAS) in 2% of cases.

AxSpA patients with FM had more pain/fatigue and a substantially lower quality-of-life than patients without FM, consistent with findings in two previous reports [7, 8]. Also, standard instruments of axSpA disease activity, that are fully (BASDAI) or partly (ASDAS) patient-reported, were significantly worse when FM was present, aligning with earlier findings [8, 38]. However, more objective inflammatory signs such as swollen joints or CRP, did not differ between axSpA patients with versus without FM in the current study. Regarding CRP, this is largely congruent with earlier findings [7, 8], although a minor difference in swollen joint count (0.26 units higher for FM versus no-FM by 66-joint index) was reported by Macfarlane et al. Since CRP is usually higher in axSpA patients with more disease activity, our findings suggest that the worse disease activity scores seen in axSpA patients with FM are possibly due to FM-induced effects on patient-reported items rather than elevated inflammation.

In the present study, axSpA patients with (versus without) comorbid FM had more often tried ≥ 1 csDMARD, and almost four times as many in the FM group had received ≥ 3 bDMARDs. Similar treatment patterns have been observed before: in one study, axSpA patients with (versus without) FM had more often received bDMARDs [7], and in another study, the FM-group had a shorter mean duration on their first TNF inhibitor and a lower retention rate after 2 years (28% versus 42%) [16]. Our results thus lend some further strength to concerns that axSpA patients with FM might more frequently initiate, switch between, and use higher numbers of DMARDs improperly. However, alternative explanations for the displayed treatment patterns, such as remaining axSpA inflammation, presence of other pain-related comorbidities, and differential healthcare utilization/physician behavior, might also play a role, and causality cannot be inferred from the cross-sectional design. Nevertheless, the task of disentangling FM symptoms from axSpA-related inflammation seems particularly challenging since enthesitis – common in axSpA [3] − can present in similar ways as widespread pain, while frequently failing to produce CRP elevation [39]. Hence, more reliable tools for detecting FM in axSpA would be valuable. Last, and notably, almost four times as many axSpA patients had ongoing opioids in the FM-group, which is generally not recommended for nociplastic pain conditions.

Half of the working-age axSpA patients with FM were employed compared to 90% without FM, with FM-positive patients constituting 35% of all non-employed patients; results congruent with low employment rates for FM patients in the general population [40]. Our findings thus suggest that FM − also as a comorbid condition in axSpA − entails large societal costs via productivity losses, and is consistent with previous reports from our group regarding the impact of subjective disease measures on work-loss in rheumatoid arthritis [41, 42]. The absence of significant differences for absenteeism/presenteeism could partly be a power issue. However, since only half of the working-age axSpA patients affected by FM were employed (and available for absenteeism/presenteeism assessment), the healthiest subsample within the FM-group might have been compared with a more unselected sample from the no-FM group. Lending further strength to this reasoning, we found a significantly higher overall activity impairment among axSpA patients with FM in analyses not restricted to working-age/dependent on employment status.

Lower pain threshold/pain tolerance, assessed with cuff pressure algometry (CPA), were both associated with presence of comorbid FM, aligning with results from CPA assessments of FM patients without inflammatory rheumatic disease [16]. TSI has been proposed as a measure to assess pain sensitization, designed to capture an increasing pain sensation during a prolonged sequence of constant pain stimulus. However, no difference in TSI was displayed between axSpA patients with versus without FM, contrasting to previous findings in patients with chronic pain syndromes such as whip-lash associated disorder [43], and lateral epicondylalgia [44]. Notably, in those studies, patients were compared with a control group recruited with the prerequisite not to have chronic pain, while in our study the comparison group without FM had an inflammatory rheumatic disease (axSpA) and some patients also widespread pain not meeting FM criteria [19].

Regarding the capacity of CPA to distinguish between FM and no-FM in axSpA, we found significant AUCs in ROC analyses: 0.77 for pain threshold and 0.79 for pain tolerance, indicating acceptable albeit not excellent discriminative ability [45]. Cut-off values using the Youden index were 24.5 kPa for pain threshold (sensitivity/specificity; 79%/70%) and 42.9 kPa for pain tolerance (sensitivity/specificity; 71%/83%). Although these values are fairly decent, the accuracy (the model’s ability to correctly classify patients as having/not having FM) was modest, with good negative predictive values (99% for pain threshold, 98% for pain tolerance), but poorer positive ones (16%/23%). Hence, for FM rule-out among axSpA patients, CPA might provide complementary information, but with limited utility for rule-in. Additionally, CPA might serve a function as a more evaluator-independent assessment tool, potentially for monitoring pain in axSpA patients over time, but longitudinal studies are needed.

### Strengths and limitations

Strengths of the current study include the population-based design and a high turn-out among eligible patients. Additionally, patients were thoroughly assessed by a predefined protocol comprising physical examinations, a rigorous imaging algorithm and comprehensive patient questionnaires, to ensure detailed classification by the modified New York and ASAS criteria for axSpA, and 1990 ACR criteria for FM. To the best of our knowledge, this was also the first study to assess pain sensitivity with CPA in axSpA patients with versus without FM.

Several limitations should be considered. First, the small FM group (*n* = 21) and large array of comparisons could entail both Type 1 errors, Type 2 errors, and limited adjustment possibilities, necessitating use of non-parametric statistics and handling of multiple comparisons. Still, remaining uncertainty warrants carefulness in the interpretation of both positive and negative results, as well as effect sizes, and larger confirmatory studies are needed. There is also possible residual confounding in the analyses, including those concerning CPA and work outcomes, potentially influencing the results. Second, the cross-sectional design does not allow for causality evaluation and hypotheses regarding directions of effects were strictly theory-based. Third, 22 patients were excluded due to missing data on items necessary for FM classification and one patient due to ongoing severe infection, although no significant differences in demographic/disease variables were seen between excluded/included patients. Fourth, although generally of minor magnitude, missing data were present, most notably for CPA with 39 patients in the study cohort lacking such data (43 for TSI). Most baseline characteristics were similar between patients with versus without CPA data (Supplementary Table S6), although patients with such data had shorter symptom duration, lower BASDAI and were more often on NSAIDS, which could potentially have affected the results. Moreover, although the population-based design is a strength, the single-centre setting may limit generalizability to axSpA cohorts with different characteristics. Finally, while the 1990 ACR criteria for FM is still a cornerstone for research classification, a revision of the 2010/2011 ACR (the 2016 ACR criteria) has been increasingly adopted, and usage of both criteria sets (1990/2016) together have been recommended for a more comprehensive evaluation [12]. Since the 2016 criteria include symptoms beyond pain such as tiredness, sleeping problems and cognitive difficulties, absence of such items may have resulted in a more one-dimensional FM assessment [46], although not necessarily affecting prevalence estimates. However, the 2016 criteria were published after start of inclusion in the SPARTAKUS cohort, and such assessments were not available for the current study. Furthermore, several other comorbidities could have symptoms overlapping those of FM, potentially impacting FM prevalence estimates and entailing patient heterogeneity. This, however, is presumably a less pronounced problem when using the 1990 criteria (as in the current study), which do not require exclusion of other disorders for fulfilment.

## Conclusion

FM is a common comorbidity in axSpA and markedly more frequent in female patients and in patients with higher BMI. Its presence was associated with several disease and health-related outcomes with higher disease activity scores, lower quality-of-life and less employment, whereas no association was seen to more objective signs of inflammation such as swollen joints and CRP. Furthermore, axSpA patients with FM had more often tried multiple bDMARDs, which may at least to some degree reflect the difficulty to differentiate between axSpA-related inflammatory pain and widespread pain due to comorbid FM, potentially resulting in DMARD overtreatment. Algometry-assessed pain threshold and pain tolerance may provide useful complementary information for FM rule-out, but less so for rule-in among axSpA patients, and can particularly add a more evaluator-independent pain assessment. Taken together, our results highlight a pronounced negative health impact of comorbid FM in axSpA and imply that its presence may entail suboptimal treatment decisions, indicating a need for earlier and more precise targeting.

## Supplementary Information

Below is the link to the electronic supplementary material.

Supplementary Material 2

## References

[1] Heidari F, Afshari M, Moosazadeh M (2017) Prevalence of fibromyalgia in general population and patients, a systematic review and meta-analysis. Rheumatol Int 37(9):1527–1539. 10.1007/s00296-017-3725-2 28447207 10.1007/s00296-017-3725-2

[2] Queiroz LP (2013) Worldwide epidemiology of fibromyalgia. Curr Pain Headache Rep 17(8):356. 10.1007/s11916-013-0356-5 23801009 10.1007/s11916-013-0356-5

[3] Zhao SS, Duffield SJ, Goodson NJ (2019) The prevalence and impact of comorbid fibromyalgia in inflammatory arthritis. Best Pract Res Clin Rheumatol 33(3):101423. 10.1016/j.berh.2019.06.005 31703796 10.1016/j.berh.2019.06.005

[4] Jones GT, Mallawaarachchi B, Shim J, Lock J, Macfarlane GJ (2020) The prevalence of fibromyalgia in axial spondyloarthritis. Rheumatol Int 40(10):1581–1591. 10.1007/s00296-020-04621-5 32556474 10.1007/s00296-020-04621-5 PMC7452944

[5] Baraliakos X, Regel A, Kiltz U, Menne HJ, Dybowski F, Igelmann M, Kalthoff L, Krause D, Saracbasi-Zender E, Schmitz-Bortz E, Braun J (2018) Patients with fibromyalgia rarely fulfil classification criteria for axial spondyloarthritis. Rheumatology (Oxford) 57(9):1541–1547. 10.1093/rheumatology/kex318 28968885 10.1093/rheumatology/kex318

[6] Moltó A, Etcheto A, Gossec L, Boudersa N, Claudepierre P, Roux N, Lemeunier L, Martin A, Sparsa L, Coquerelle P, Soubrier M, Perrot S, Dougados M (2018) Evaluation of the impact of concomitant fibromyalgia on TNF alpha blockers’ effectiveness in axial spondyloarthritis: results of a prospective, multicentre study. Ann Rheum Dis 77(4):533–540. 10.1136/annrheumdis-2017-212378 29183878 10.1136/annrheumdis-2017-212378

[7] Macfarlane GJ, Barnish MS, Pathan E, Martin KR, Haywood KL, Siebert S, Packham J, Atzeni F, Jones GT (2017) Co-Occurrence and Characteristics of Patients With Axial Spondyloarthritis Who Meet Criteria for Fibromyalgia: Results From a UK National Register. Arthritis Rheumatol 69(11):2144–2150. 10.1002/art.40185 28622461 10.1002/art.40185

[8] Sayın S, Yurdakul FG, Sivas F, Bodur H (2020) Is fibromyalgia frequency increasing in axial spondyloarthritis? Association with fibromyalgia and biological therapies. Rheumatol Int 40(11):1835–1841. 10.1007/s00296-020-04670-w 32767083 10.1007/s00296-020-04670-w

[9] Woolf CJ (2011) Central sensitization: Implications for the diagnosis and treatment of pain. Pain 152(3):S2–S15. 10.1016/j.pain.2010.09.030 20961685 10.1016/j.pain.2010.09.030 PMC3268359

[10] Sarzi-Puttini P, Giorgi V, Marotto D, Atzeni F (2020) Fibromyalgia: an update on clinical characteristics, aetiopathogenesis and treatment. Nat Rev Rheumatol 16(11):645–660. 10.1038/s41584-020-00506-w 33024295 10.1038/s41584-020-00506-w

[11] Benebo FO, Lukic M, Jakobsen MD, Braaten TB (2023) Lifestyle risk factors of self-reported fibromyalgia in the Norwegian Women and Cancer (NOWAC) study. BMC Public Health 23(1):1967. 10.1186/s12889-023-16773-7 37821848 10.1186/s12889-023-16773-7 PMC10566054

[12] Sarzi-Puttini P, Giorgi V, Atzeni F, Gorla R, Kosek E, Choy EH, Bazzichi L, Häuser W, Ablin JN, Aloush V, Buskila D, Amital H, Da Silva JAP, Perrot S, Morlion B, Polati E, Schweiger V, Coaccioli S, Varrassi G, Di Franco M, Torta R, Øien Forseth KM, Mannerkorpi K, Salaffi F, Di Carlo M, Cassisi G, Batticciotto A (2021) Fibromyalgia position paper. Clin Exp Rheumatol 39(Suppl 130):186–193. 10.55563/clinexprheumatol/i19pig 34001303 10.55563/clinexprheumatol/i19pig

[13] Wallace DJ, Gavin IM, Karpenko O, Barkhordar F, Gillis BS (2015) Cytokine and chemokine profiles in fibromyalgia, rheumatoid arthritis and systemic lupus erythematosus: a potentially useful tool in differential diagnosis. Rheumatol Int 5(6):991–996. 10.1007/s00296-014-3172-2 10.1007/s00296-014-3172-2 PMC4435905 25377646

[14] Krock E, Morado-Urbina CE, Menezes J, Hunt MA, Sandström A, Kadetoff D, Tour J, Verma V, Kultima K, Haglund L, Meloto CB, Diatchenko L, Kosek E, Svensson CI (2023) Fibromyalgia patients with elevated levels of anti-satellite glia cell immunoglobulin G antibodies present with more severe symptoms. Pain 164(8):1828–1840. 10.1097/j.pain.0000000000002881 36943275 10.1097/j.pain.0000000000002881 PMC10348624

[15] Coskun Benlidayi I (2020) Fibromyalgia interferes with disease activity and biological therapy response in inflammatory rheumatic diseases. Rheumatol Int 40:849–858. 10.1007/s00296-019-04506-2 31900502 10.1007/s00296-019-04506-2

[16] Bello N, Etcheto A, Béal C, Dougados M, Moltó A (2016) Evaluation of the impact of fibromyalgia in disease activity and treatment effect in spondyloarthritis. Arthritis Res Ther 18:42. 10.1186/s13075-016-0943-z 26860612 10.1186/s13075-016-0943-z PMC4748456

[17] Polianskis R, Graven-Nielsen T, Arendt-Nielsen L (2001) Computer-controlled pneumatic pressure algometry–a new technique for quantitative sensory testing. Eur J Pain 5(3):267–277. 10.1053/eujp.2001.0245 11558982 10.1053/eujp.2001.0245

[18] Jespersen A, Dreyer L, Kendall S, Graven-Nielsen T, Arendt-Nielsen L, Bliddal H, Danneskiold-Samsoe B (2007) Computerized cuff pressure algometry: A new method to assess deep-tissue hypersensitivity in fibromyalgia. Pain 131(1):57–62. 10.1016/j.pain.2006.12.012 17257757 10.1016/j.pain.2006.12.012

[19] Olofsson T, Lindqvist E, Mogard E, Andréasson K, Marsal J, Geijer M, Kristensen LE, Wallman JK (2019) Elevated faecal calprotectin is linked to worse disease status in axial spondyloarthritis: results from the SPARTAKUS cohort. Rheumatology (Oxford) 58(7):1176–1187. 10.1093/rheumatology/key427 30649509 10.1093/rheumatology/key427

[20] Wolfe F, Smythe HA, Yunus MB, Bennett RM, Bombardier C, Goldenberg DL, Tugwell P, Campbell SM, Abeles M, Clark P et al (1990) The American College of Rheumatology 1990 Criteria for the Classification of Fibromyalgia. Report of the Multicenter Criteria Committee. Arthritis Rheum 33(2):160–172. 10.1002/art.1780330203 2306288 10.1002/art.1780330203

[21] Mogard E, Olofsson T, Bergman S, Bremander A, Kristensen LE, Olsen JK, Wallman JK, Lindqvist E (2021) Chronic Pain and Assessment of Pain Sensitivity in Patients With Axial Spondyloarthritis: Results From the SPARTAKUS Cohort. J Rheumatol 48(11):1672–1679. 10.3899/jrheum.200872 33323532 10.3899/jrheum.200872

[22] Rudwaleit M, van der Heijde D, Landewé R, Listing J, Akkoc N, Brandt J, Braun J, Chou CT, Collantes-Estevez E, Dougados M, Huang F, Gu J, Khan MA, Kirazli Y, Maksymowych WP, Mielants H, Sørensen IJ, Ozgocmen S, Roussou E, Valle-Oñate R, Weber U, Wei J, Sieper J (2009) The development of Assessment of SpondyloArthritis international Society classification criteria for axial spondyloarthritis (part II): validation and final selection. Ann Rheum Dis 68(6):777–783. 10.1136/ard.2009.108233 19297344 10.1136/ard.2009.108233

[23] van der Linden S, Valkenburg HA, Cats A (1984) Evaluation of diagnostic criteria for ankylosing spondylitis. A proposal for modification of the New York criteria. Arthritis Rheum 27(4):361–368. 10.1002/art.1780270401 6231933 10.1002/art.1780270401

[2] Socialstyrelsen (2018) Nationella riktlinjer för prevention och behandling vid ohälsosamma levnadsvanor. https://www.socialstyrelsen.se/globalassets/sharepoint-dokument/artikelkatalog/nationella-riktlinjer/2018-6-24.pdf. 2018-6-24

[25] Lukas C, Landewé R, Sieper J, Dougados M, Davis J, Braun J, van der Linden S, van der Heijde D (2009) Development of an ASAS-endorsed disease activity score (ASDAS) in patients with ankylosing spondylitis. Ann Rheum Dis 68(1):18–24. 10.1136/ard.2008.094870 18625618 10.1136/ard.2008.094870

[26] Garrett S, Jenkinson T, Kennedy LG, Whitelock H, Gaisford P, Calin A (1994) A new approach to defining disease status in ankylosing spondylitis: the Bath Ankylosing Spondylitis Disease Activity Index. J Rheumatol 21(12):2286–2291 7699630

[27] Calin A, Garrett S, Whitelock H, Kennedy LG, O’Hea J, Mallorie P, Jenkinson T (1994) A new approach to defining functional ability in ankylosing spondylitis: the development of the Bath Ankylosing Spondylitis Functional Index. J Rheumatol 21(12):2281–2285 7699629

[28] Jenkinson TR, Mallorie PA, Whitelock HC, Kennedy LG, Garrett SL, Calin A (1994) Defining spinal mobility in ankylosing spondylitis (AS). The Bath AS Metrology Index. J Rheumatol 21(9):1694–1698 7799351

[29] Wallman JK, Eriksson JK, Nilsson J, Olofsson T, Kristensen LE, Neovius M, Geborek P (2016) Costs in Relation to Disability, Disease Activity, and Health-related Quality of Life in Rheumatoid Arthritis: Observational Data from Southern Sweden. J Rheumatol 43(7):1292–1299. 10.3899/jrheum.150617 27252420 10.3899/jrheum.150617

[30] Åstrand P (1965) Work tests with the bicycle ergometer. Gymnastik- och Idrottshögskolan, Stockholm

[31] Reilly MC, Gooch KL, Wong RL, Kupper H, van der Heijde D (2010) Validity, reliability and responsiveness of the Work Productivity and Activity Impairment Questionnaire in ankylosing spondylitis. Rheumatology (Oxford) 49(4):812–819. 10.1093/rheumatology/kep457 20100797 10.1093/rheumatology/kep457

[32] Kvistgaard Olsen J, Fener DK, Waehrens EE, Wulf Christensen A, Jespersen A, Danneskiold-Samsøe B, Bartels EM (2017) Reliability of Pain Measurements Using Computerized Cuff Algometry: A DoloCuff Reliability and Agreement Study. Pain Pract 17(6):708–717. 10.1111/papr.12514 27611494 10.1111/papr.12514

[33] Wolfe F, Clauw DJ, Fitzcharles MA, Goldenberg DL, Häuser W, Katz RS, Mease P, Russell AS, Russell IJ, Winfield JB (2011) Fibromyalgia criteria and severity scales for clinical and epidemiological studies: a modification of the ACR Preliminary Diagnostic Criteria for Fibromyalgia. J Rheumatol 38(6):1113–1122. 10.3899/jrheum.100594 21285161 10.3899/jrheum.100594

[34] Wolfe F, Clauw DJ, Fitzcharles MA, Goldenberg DL, Häuser W, Katz RL, Mease PJ, Russell AS, Russell IJ, Walitt B (2016) 2016 Revisions to the 2010/2011 fibromyalgia diagnostic criteria. Semin Arthritis Rheum 46(3):319–329. 10.1016/j.semarthrit.2016.08.012 27916278 10.1016/j.semarthrit.2016.08.012

[35] Jones GT, Atzeni F, Beasley M, Flüß E, Sarzi-Puttini P, Macfarlane GJ (2015) The prevalence of fibromyalgia in the general population: a comparison of the American College of Rheumatology 1990, 2010, and modified 2010 classification criteria. Arthritis Rheumatol 67(2):568–575. 10.1002/art.38905 25323744 10.1002/art.38905

[36] Wach J, Letroublon MC, Coury F, Tebib JG (2016) Fibromyalgia in Spondyloarthritis: Effect on Disease Activity Assessment in Clinical Practice. J Rheumatol 43(11):2056–2063. 10.3899/jrheum.160104 27633820 10.3899/jrheum.160104

[37] Creed F (2020) A review of the incidence and risk factors for fibromyalgia and chronic widespread pain in population-based studies. Pain 161(6):1169–1176. 10.1097/j.pain.0000000000001819 32040078 10.1097/j.pain.0000000000001819

[38] Duffield SJ, Miller N, Zhao S, Goodson NJ (2018) Concomitant fibromyalgia complicating chronic inflammatory arthritis: a systematic review and meta-analysis. Rheumatology 57(8):1453–1460. 10.1093/rheumatology/key112 29788461 10.1093/rheumatology/key112 PMC6055651

[39] Marchesoni A, De Marco G, Merashli M, McKenna F, Tinazzi I, Marzo-Ortega H, McGonagle DG (2018) The problem in differentiation between psoriatic-related polyenthesitis and fibromyalgia. Rheumatology (Oxford) 57(1):32–40. 10.1093/rheumatology/kex079 28387854 10.1093/rheumatology/kex079

[40] Palstam A, Mannerkorpi K (2017) Work Ability in Fibromyalgia: An Update in the 21st Century. Curr Rheumatol Rev 13(3):180–187. 10.2174/1573397113666170502152955 28464770 10.2174/1573397113666170502152955 PMC5759171

[41] Olofsson T, Petersson IF, Eriksson JK, Englund M, Simard JF, Nilsson J, Geborek P, Jacobsson LT, Askling J, Neovius M (2014) Predictors of work disability during the first 3 years after diagnosis in a national rheumatoid arthritis inception cohort. Ann Rheum Dis 73(5):845–853. 10.1136/annrheumdis-2012-202911 23520035 10.1136/annrheumdis-2012-202911

[42] Olofsson T, Söderling JK, Gülfe A, Kristensen LE, Wallman JK (2018) Patient-Reported Outcomes Are More Important Than Objective Inflammatory Markers for Sick Leave in Biologics-Treated Patients With Rheumatoid Arthritis. Arthritis Care Res (Hoboken) 70(11):1712–1716. 10.1002/acr.23619 29885037 10.1002/acr.23619

[43] Lemming D, Graven-Nielsen T, Sörensen J, Arendt-Nielsen L, Gerdle B (2012) Widespread pain hypersensitivity and facilitated temporal summation of deep tissue pain in whiplash associated disorder: an explorative study of women. J Rehabil Med 44(8):648–657 22729792 10.2340/16501977-1006

[44] Jespersen A, Amris K, Graven-Nielsen T, Arendt-Nielsen L, Bartels EM, Torp-Pedersen S, Bliddal H, Danneskiold-Samsoe B (2013) Assessment of pressure-pain thresholds and central sensitization of pain in lateral epicondylalgia. Pain Med 14(2):297–304. 10.1111/pme.12021 23279601 10.1111/pme.12021

[45] Hosmer DW, Lemeshow S (2013) Applied Logistic Regression. Wiley, New Jersey

[46] Glanlorenco AC, Costa V, Fabris–Moraes W, Menacho M, Alves LC, Martinez–Magallanes D, Fregnl F (2024) Cluster analysis in fibromyalgia: a systematic review. Rheum Int 44:2389–2402. 10.1007/s00296-024-05616-2 10.1007/s00296-024-05616-2 38748219

