# Supplementary material for: Axial spondyloarthritis patients with comorbid fibromyalgia feel worse, work less and more often try multiple biological therapies: results from a population-based, cross-sectional study investigating the discriminative capacity of pressure algometry
Source: Rheumatol Int. 2026 May 23;46(6):99. doi: 10.1007/s00296-026-06140-1 (PMC13198514; doi:10.1007/s00296-026-06140-1)
Supplement: Supplementary file 1 — Supplementary Material 2 [file 296_2026_6140_MOESM2_ESM.docx]

**SUPPLEMENTARY MATERIAL**

Axial Spondyloarthritis Patients with Comorbid Fibromyalgia Feel Worse, Work Less and More Often Try Multiple Biological Therapies – Results from a Population-Based, Cross-Sectional Study Investigating the Discriminative Capacity of Pressure Algometry

Annie Brink, Elisabeth Mogard, Elisabet Lindqvist, Jonas Sagard, Carmen Roseman, Mats Geijer, Jack Kvistgaard Olsen, Johan K. Wallman, Tor Olofsson

**Table of Contents Page**

**Supplementary Methods** ……………………………………………………………….. **2-4**

**Supplementary Table S1** ………………………………………………………………… **5**

**Supplementary Table S2** ………………………………………………………………… **6**

**Supplementary Table S3** ………………………………………………………………… **7**

**Supplementary Table S4** ………………………………………………………………… **8**

**Supplementary Table S5** ………………………………………………………………… **9**

**Supplementary Table S6 ………………………………………………………………. 10**

**Supplementary Figure S1** ………………………………………………………………**.. 11**

**References …………………….**………………………………………………………………**. 12**

| **SUPPLEMENTARY METHODS**  **Additional information regarding treatments**  *DMARDs*  In the current study, the conventional synthetic disease modifying anti-rheumatic drugs (csDMARDs) that axSpA patients were taking/had received earlier include (total number in FM/no FM group): methotrexate (12/69), sulfasalazine (2/9), hydroxychloroquine (2/9), azathioprin (2/6), gold therapy (2/3), mycophenolate mofetil (1/1), and klorambucil (0/1). The biologic DMARDs (bDMARDs) include (total number in FM/no FM group): etanercept (8/56), adalimumab (5/42), infliximab (4/35), certolizumab pegol (5/31), golimumab (3/11), secukinumab (2/2), tocilizumab (1/3), and rituximab (0/1). Beyond csDMARDs/bDMARDs, 1 patient without FM was taking apremilast. The variable ”any DMARDs” include csDMARDs, bDMARDs and apremilast.  *Other treatments*  The variable “other antidepressants” include (total number in the FM/no FM group) selective serotonin reuptake inhibitors (1/22) (fluoxetine, citalopram, paroxetine, sertraline, escitalopram, fluvoxamine) and serotonin-norepinephrine reuptake inhibitors (3/10) (mianserin, mirtazapine, bupropion, venlafaxine, duloxetine).  **Additional information on lifestyle factors**  Unhealthy (high-risk) alcohol use was defined as weekly standard unit consumption >14/>9 for men/women, and unhealthy (insufficient) physical activity as <150 minutes weekly exercise of at least moderate intensity.  **Additional information on work and activity measures**  Work productivity and activity limitation were assessed with the Work Productivity and Activity Impairment Questionnaire: General Health (WPAI:GH). The WPAI:GH generates four scores: percentage of absenteeism (time missed from work due to poor health), percentage of presenteeism (productivity impairment at work due to poor health), percentage of overall work impairment (composite measure of both absenteeism and presenteeism) and percentage of activity impairment (impairment in activities outside work). Measures of work ability were analyzed in people of working age in Sweden (18-65 years), while activity impairment was assessed in all age-groups.  **Additional information on pain assessments using the DoloCuff algometry device**  The DoloCuff (software version 2.0.5.1) is a computerized pneumatic cuff pressure algometry device (CPA), consisting of an inflatable textile tourniquet cuff with two chambers (reference number 20-50-727; VMB medizin-technik Gmbh, Sulz, Germany) and a computer-controlled air compressor. The patient reports pain continuously during the examination using a hand-held electronic 10-cm VAS. Examinations were performed in supine position in a temperate, closed and noise-reduced room, and the cuff was fitted to the widest part of the dominant legs’ gastrocnemius muscle. Three sequences were performed; a short sequence used to introduce the assessment to the patient, an auto-sequence consisting of 3 short sequences with 3 minutes rest between measurements (continuous inflation of the cuff with 1.0 kPa/second), and a long sequence of 10 minutes or of the time until pain became intolerable (constant cuff pressure [CP] used). The auto sequence was used to measure: 1) pain threshold (PTh), defined as the pressure (in kPa) when the sensation of strong pressure first transitioned to being painful, and 2) pain tolerance (PTol), defined as the pressure (in kPa) when the pressure of the cuff had to be stopped due to intolerable pain. The mean values for PTh and PTol were then calculated from the 3 auto-sequences. The degree of temporal summation (TS) was assessed during the long sequence. The CP used was determined by each patient’s individual values on PTh and PTol: $CP= {PTh}_{mean}+\left( 0.5*\left[ {PTol}_{mean}-{PTh}_{mean} \right] \right)$.^1^ A temporal summation index (TSI) was calculated using the highest VAS pain rating measured during the long sequence (VASmax, 0–10), the VAS pain at the end of the sequence (VASend, 0–10), and the stimulation time (t): $TSI= log(\left[ \frac{{VAS}_{end}}{{VAS}_{max}} \right]*\left[ \frac{10}{t} \right]*{VAS}_{end})$.^2^ Higher TSI scores are designed to indicate more temporal summation. Patients were requested to pause analgesics 48 hours before the investigation.  **Additional information regarding statistics**  Demographics, axSpA phenotype, and lifestyle factors – hypothesized to constitute potential risk factors of FM based on literature review and previous experience – were analyzed by logistic regression with FM (yes/no) as dependent variable. Significant variables were subsequently introduced into a multivariate model (checking for collinearity by Spearman’s/Pearson’s correlation tests, ascertaining that covariates did not display an r > 0.4/<-0.4 with each other). Linearity of the logit was checked in regression models with continuous independent variables. Variables rather hypothesized to be impacted by FM, were analyzed with FM (yes/no) as an independent variable, using linear regression (continuous outcomes) and logistic regression (dichotomous outcomes); univariately and adjusted for age and sex (adjustment performed for dichotomous outcomes provided there were at least 10 events in the smallest group of the studied outcome). 95% confidence intervals (95% CI) were obtained via non-parametric, bias-corrected accelerated bootstrapping using 1000 samples for skewed variables (residuals normality-tested by Shapiro-Wilk tests). ROC-curve analysis was adopted to explore diagnostic potential of CPA-assessed pain sensitivity measures (for distinguishing FM from no FM), including bootstrap-generated 95% confidence intervals calculated for AUC estimates to account for the limited sample size and sample imbalance, while utilizing the Youden index to determine cut-off points for each measure. Due to the limited sample size, no train/test split was applied and all analyses were performed on the full study sample. To account for multiple comparisons, the Benjamini-Hochberg procedure (BH) was applied, controlling for a false discovery rate (FDR) of 0.05. In doing so , tests were regarded as analysis entities based on underlying null hypothesis, population studied and regression model used. BH was not applied if the number of tests within an entity was less than ten. |
| --- |

**Additional comorbidity data**

Data on additional potentially pain-influencing comorbidites were retrieved through ICD-codes from our administrative register, RSVD (Region Skånes Vårddatabas),which covers specialized in- and outpatient care, as well as primary care. The following comorbidities were collected: diabetes (ICD-codes E10-E14 ); thyroid disease (E03, E05, E06, O905); peripheral neuropathies (G50-G64); vitamin B12 deficiency (D51); depression (F32-F34); anxiety disorders (F40-F42, F430-F432, F438W, F439); requring a diagnosis code at one occasion to count into the respective categories. Data were retrieved for the 10-year period preceding the date of inclusions in the SPARTAKUS cohort (when the pressure algometry evaluation was also performed). Analysis was restricted to patients residing in Skåne county during the entire period (n=222/243 included axSpA patients).

**Supplementary Table S1.** Characteristics of SPARTAKUS patients included in the current study and excluded patients.

|  | **Included (n=243)** | **Excluded (n=23)** | **P-value** |
| --- | --- | --- | --- |
| Age, years | 51 (13) | 53 (15) | 0.457 |
| Male sex, n (%) | 130 (53%) | 13 (57%) | 0.781 |
| Symptom duration, years | 25 (14) | 26 (14) | 0.693 |
| BMI, kg/m2 | 26 (5) | 27 (6) | 0.965 |
| r-axSpA, n (%) | 165 (68%) | 15 (65%) | 0.793 |
| HLA-B27 positivity, n (%) | 209 (86%) | 18 (78%) | 0.345 |
| SJC (of 66) | 0.2 (0.7) | 0.1 (0.5) | 0.741 |
| TJC (of 68) | 3.9 (6.1) | 3.4 (7.2) | 0.401 |
| CRP, mg/L | 3.4 (4.6) | 7.8 (19) | 0.410 |
| ASDAS-CRP | 1.8 (0.9) | 2.1 (1.1) | 0.381 |
| BASDAI | 3.0 (2.2) | 3.1 (2.3) | 0.882 |
| NSAIDs, ongoing, n (%) | 154 (63%) | 15 (68%) | 0.653 |
| bDMARDs, ongoing, n (%) | 103 (42%) | 10 (43%) | 0.919 |
| csDMARDs, ongoing, n (%) | 48 (20%) | 6 (26%) | 0.429 |

All results are presented as mean (SD) unless otherwise indicated. Group comparisons by Student’s t-test, Mann-Whitney U-test, Chi-square test, and Fisher’s exact test, as appropriate. Missing data: symptom duration 1, HLA-B27 status 1, SJC 5, TJC 5, CRP 1, ASDAS-CRP 6, BASDAI 7. ASDAS-CRP, ankylosing spondylitis disease activity score with CRP; AxSpA, axial spondyloarthritis; BASDAI, Bath ankylosing spondylitis disease activity index; bDMARDs, biologic disease-modifying antirheumatic drugs; cDMARDs, conventional synthetic DMARDs; CRP, C-reactive protein; r-axSpA, radiographic axSpA; SJC, swollen joint count; TJC, tender joint count. P-values <0.05 considered significant.

**Supplementary Table S2.** Background factors associated with presence of fibromyalgia in axSpA patients.

|  | **Univariate** | | **Multivariate*** | |
| --- | --- | --- | --- | --- |
|  | *OR (95% CI)* | *P value* | *OR (95% CI)* | *P value* |
| Age, per 10-year increase | 1.07 (0.76 to 1.51) | 0.709 |  |  |
| Age (≥50y vs. <50y) | 1.81 (0.72 to 4.54) | 0.206 |  |  |
| Symptom duration, per 10-year increase | 1.11 (0.81 to 1.53) | 0.515 |  |  |
| Symptom duration (≥25y vs <25y) | 1.00 (0.41 to 2.46) | 0.992 |  |  |
| Female sex (yes vs. no) | 12.9 (2.9 to 56.9) | *<0.001* | 13.4 (3.0 to 61.1) | *<0.001* |
| axSpA subtype (nr-axSpA vs. r-axSpA) | 0.83 (0.31 to 2.34) | 0.717 |  |  |
| HLA-B27 (no vs. yes) | 2.87 (1.03 to 8.04) | *0.044* | 2.05 (0.66 to 6.40) | 0.218 |
| BMI, per 5-unit increase | 2.02 (1.29 to 3.17) | *0.002* | 2.17 (1.34 to 3.51) | *0.002* |
| BMI (≥25 vs. <25) | 5.19 (1.49 to 18.13) | *0.01* | 8.18 (2.20 to 30.4) | *0.002* |
| Never smoker (yes vs. no) | 0.59 (0.24 to 1.46) | 0.257 |  |  |
| High-risk alcohol use (yes vs. no) | 0.98 (0.27 to 3.52) | 0.973 |  |  |
| Unhealthy physical activity (yes vs. no) | 2.00 (0.80 to 5.01) | 0.137 |  |  |
| Ever peripheral arthritis (yes vs. no) | 1.22 (0.49 to 3.01) | 0.668 |  |  |
| Ever dactylitis (yes vs. no) | 1.52 (0.41 to 5.56) | 0.531 |  |  |
| Ever uveitis (yes vs. no) | 0.98 (0.40 to 2.43) | 0.973 |  |  |
| Psoriasis (yes vs. no) | 1.78 (0.48 to 6.60) | 0.388 |  |  |
| IBD (yes vs. no) | 1.36 (0.29 to 6.34) | 0.699 |  |  |

Odds ratios (ORs) from logistic regression with P-values <0.05 considered significant. High-risk alcohol use: weekly consumption of >14 standard units (men) and >9 standard units (women). Unhealthy physical activity: <150 minutes of weekly exercise of at least moderate intensity. Cut-offs for alcohol use/physical activity according to recommendations from the Swedish National Board of Health and Welfare. Musculoskeletal and extra-musculoskeletal manifestations are categorized as yes (ever) or no (never). Missing data: symptom duration 1, HLA-B27 status 1, smoking status 14, alcohol use 9, physical activity 9. AxSpA, axial spondyloarthritis; BMI, body mass index; HLA-B27, human leukocyte antigen B27; IBD, inflammatory bowel disease; nr-axSpA, non-radiographic axSpA; r-axSpA, radiographic axSpA; vs, versus; y, years. *Univariately significant variables were introduced into a multivariate analysis, checking for collinearity by Spearman’s/Pearson’s correlation tests to ascertain that such covariates did not display an r >0.4/<-0.4 with each other. BMI was entered as a continuous variable when estimating female sex and HLA-B27.

**Supplementary Table S3.** Associations between comorbid fibromyalgia and pharmacological treatments.

|  |  |  | **Univariate** | | **Age- and sex adjusted** | |
| --- | --- | --- | --- | --- | --- | --- |
|  | *FM*  *(n=21)* | *No FM (n=222)* | *OR (95% CI)* | *P value* | *OR (95% CI)* | *P value* |
| Ongoing therapy | | | | | | |
| Any DMARDs | 14 (67%) | 112 (50%) | 1.9 (0.7 to 4.8) | 0.198 | 2.0 (0.7 to 5.2) | 0.179 |
| csDMARDs | 8 (38%) | 40 (18%) | 2.8 (1.1 to 7.2) | *0.033* | 2.4 (0.9 to 6.4) | 0.085 |
| bDMARDs | 10 (48%) | 91 (41%) | 1.3 (0.5 to 3.1) | 0.612 | 1.5 (0.6 to 3.9) | 0.388 |
| NSAIDs | 10 (48%) | 144 (65%) | 0.5 (0.2 to 1.2) | 0.123 | 0.4 (0.2 to 1.0) | 0.056 |
| Paracetamol | 19 (90%) | 108 (49%) | 10.0 (2.3 to 43.7) | *0.002* | *6.1 (1.4 to 27.5)* | *0.019* |
| Any opioids | 13 (62%) | 35 (16%) | 8.5 (3.3 to 22.1) | *<0.001* | *10.3 (3.7 to 29.1)* | *<0.001*^†^ |
| Tramadol | 5 (24%) | 14 (6%) | 4.6 (1.5 to 14.5) | *0.008* | *3.9 (1.1 to 13.2)* | *0.030* |
| Codein | 3 (14%) | 22 (10%) | 1.5 (0.4 to 5.6) | 0.531 | 1.6 (0.4 to 6.2) | 0.525 |
| Other opioids | 6 (29%) | 4 (2%) | 21.5 (5.5 to 84.5) | *<0.001* | *30.6 (5.3 to 176.5)* | *<0.001*^†^ |
| Tricyclic antidepressants | 6 (29%) | 5 (2%) | 17.4 (4.7 to 63.5) | *<0.001* | *12.0 (3.0 to 48.7)* | *<0.001*^†^ |
| Other antidepressants* | 4 (19%) | 30 (14%) | 1.5 (0.5 to 4.8) | 0.487 | 1.4 (0.4 to 4.7) | 0.589 |
| DMARDs ever |  |  |  |  |  |  |
| Any DMARDs (≥1) | 19 (90%) | 151 (68%) | 4.5 (1.0 to 19.7) | *0.048* | *5.2 (1.2 to 23.7)* | *0.032* |
| Any DMARDs (≥2) | 15 (71%) | 91 (41%) | 3.5 (1.3 to 9.4) | *0.012* | *3.5 (1.3 to 9.7)* | *0.016* |
| Any DMARDs (≥3) | 7 (33%) | 49 (22%) | 1.7 (0.7 to 4.5) | 0.268 | 1.7 (0.6 to 4.7) | 0.296 |
| csDMARDs (≥1) | 17 (81%) | 101 (45%) | 5.1 (1.7 to 15.6) | *0.004* | *5.4 (1.7 to 17.2)* | *0.004*^†^ |
| csDMARDs (≥2) | 6 (29%) | 37 (17%) | 2 (0.7 to 5.5) | 0.179 | 1.9 (0.7 to 5.6) | 0.226 |
| csDMARDs (≥3) | 2 (10%) | 8 (4%) | 2.8 (0.6 to 14.2) | 0.210 | 4.3 (0.7 to 28.1) | 0.131 |
| bDMARDs (≥1) | 14 (67%) | 118 (53%) | 1.8 (0.7 to 4.5) | 0.240 | 2.3 (0.8 to 6.2) | 0.103 |
| bDMARDs (≥2) | 7 (33%) | 43 (19%) | 2.0 (0.8 to 5.3) | 0.153 | 1.7 (0.6 to 4.8) | 0.283 |
| bDMARDs (≥3) | 4 (19%) | 12 (5%) | 4.1 (1.2 to 14.2) | *0.025* | *7.3 (1.6 to 32.4)* | *0.009*^†^ |

P-values <0.05 considered significant. Odds ratios (ORs) from logistic regression. No missing data in FM group. Missing data in the no FM group: paracetamol 1, any opioids 3, other opioids 3. csDMARDs, conventional synthetic DMARDs; bDMARDs, biologic DMARDs; DMARDs, disease-modifying antirheumatic drugs; NSAIDs, nonsteroidal anti-inflammatory drugs. *Including selective serotonin reuptake inhibitors (SSRIs) and serotonin-norepinephrine reuptake inhibitors (SNRIs). Age- and sex-adjustment was carried out provided there were ≥10 events in the smallest group of the studied outcome variable. (†) Indicates significance also after applying the Benjamini-Hochberg procedure.

**Supplementary Table S4.** Associations between comorbid fibromyalgia and work/activity measures in axSpA patients.

|  |  | **Mean (SD)** | | **Univariate** | | **Age- and sex adjusted** | |
| --- | --- | --- | --- | --- | --- | --- | --- |
| *Working-age patients*  *(18-65y)*  *n=203* |  | *FM*  *(n =19)* | *No FM*  *(n = 184)* | *OR (95% CI)* | *P value* | *OR (95% CI)* | *P value* |
|  | Current Work,  n (%) | 10 (48) | 167 (91) | 0.11 (0.04 to 0.32) | <0.001 | 0.08 (0.02 to 0.26) | <0.001 |
|  |  |  | | *ß (95% CI)* | *P value* | *ß (95% CI)* | *P value* |
|  | Absenteeism  (0-100) | 16 (37) | 8 (22) | 8 (-10 to 45) | 0.576 | 6 (-14 to 37) | 0.724 |
|  | Presenteeism  (0-100) | 31 (25) | 24 (28) | 8 (-9 to 25) | 0.414 | 3 (-15 to 25) | 0.710 |
|  | Overall work impairment (0-100) | 44 (35) | 27 (31) | 18 (-8 to 45) | 0.177 | 11 (-17 to 39) | 0.401 |
| *All patients (18-80y) n=243* |  | *FM*  *(n =21)* | *No FM*  *(n = 222)* | *ß (95% CI)* | *P value* | *ß (95% CI)* | *P value* |
|  | Activity impairment  (0-100) | 67 (25) | 34 (29) | 33 (21 to 44) | <0.001 | 28 (16 to 40) | 0.002 |

Variables assessed by the Work Productivity and Activity Impairment – General Health Questionnaire (WPAI-GH). Presented as mean (SD), unless otherwise indicated. Odds ratios (ORs) from logistic regression and mean differences (ß) from linear regression. Age- and sex-adjustment was performed for analyses with continual outcomes and for dichotomous outcomes provided there was ≥10 events in the smallest group of the studied outcome. Absenteeism=time missed from work due to poor health (percentage); presenteeism=productivity impairment at work due to poor health (percentage); overall work impairment=composite measure of both absenteeism and presenteeism (percentage); activity impairment=overall impairment in activities outside work (percentage). Absenteeism, presenteeism and overall work impairment could only be assessed in employed patients, whereas activity impairment was assessed in all patients. Missing data in working-age patients (FM/no FM): current work, 0/1; absenteeism, 3/14; presenteeism, 3/5; overall work impairment, 3/14. Missing data in all patients (FM/no FM): activity impairment, 1/4. AxSpA, axial spondyloarthritis; CI, confidence interval; FM, fibromyalgia; SD, standard deviation; y, years.

**Supplementary Table S5.** Associations between algometry-assessed pain sensitivity measures and presence of comorbid fibromyalgia in axSpA patients.

|  | **Univariate** | | | **Age- and sex-adjusted** | | **Adjusted for other comorbidities and therapies*** | |  |
| --- | --- | --- | --- | --- | --- | --- | --- | --- |
|  |  |  |  |  |  |  |  |  |
|  | OR (95% CI) | P Value | | OR (95% CI) | P Value | OR (95% CI) | P Value |  |
| Pain Threshold (kPa, per 10-unit decrease) | 2.64 (1.35-5.18) | | 0.005 | 2.08 (1.03-4.23) | 0.042 | 2.46 (1.22-4.97 | 0.012 |  |
|  |  |  |  |  |  |  |  |  |
| Pain Tolerance (kPa, per 10-unit decrease) | 1.68 (1.18-2.38) | | 0.004 | 1.42 (1.01-1.99) | 0.046 | 1.77 (1.16-2.69) | 0.031 |  |
|  |  |  |  |  |  |  |  |  |
| TSI | 0.83 (0.43-1.63 | | 0.596 | 0.63 (0.25-1.62) | 0.336 | 1.03 (0.57-1.89) | 0.921 |  |
|  |  |  |  |  |  |  |  |  |

P-values <0.05 considered significant. Odds ratios (ORs) from logistic regression models, unadjusted and

adjusted for age and sex, as well as for other comorbidities and therapies. Patients who had not managed to pause analgesics 48 hours prior to the CPA examination, as requested, were still included (2 patients with FM, 8 patients without FM). Missing data (FM/no-FM): pain threshold, 7/32; pain tolerance, 7/32; TSI, 7/37. AxSpA, axial spondyloarthritis; FM, fibromyalgia; TSI; temporal summation index. *Other comorbidities (with possible impact on pain perception): diabetes, thyroid disease, peripheral neuropathies, vitamin B12 deficiency, depression and anxiety disorders (by ICD-10 codes, see Supplementary Methods above for retrieval methodology). Therapies potentially influencing pain perception: anti-depressants and opioids (ongoing).

**Supplementary Table S6.** Characteristics of SPARTAKUS patients with versus without computerized pressure algometry (CPA) data.

|  | **CPA (n=204)** | **No CPA (n=39)** | **P-value** |
| --- | --- | --- | --- |
| Age, years | 50 (12) | 55 (16) | 0.068 |
| Male sex, n (%) | 105 (51%) | 25 (64%) | 0.147 |
| Symptom duration, years | 24 (13) | 30 (16) | 0.028 |
| BMI, kg/m2 | 26 (5) | 26 (5) | 0.957 |
| r-axSpA, n (%) | 136 (67%) | 29 (74%) | 0.346 |
| HLA-B27 positivity, n (%) | 175 (86%) | 34 (87%) | 1.000 |
| SJC (of 66) | 0.2 (0.7) | 0.1 (0.5) | 0.449 |
| TJC (of 68) | 3.5 (5.6) | 7.8 (6.0) | 0.063 |
| CRP, mg/L | 3.4 (4.8) | 3.0 (3.7) | 0.640 |
| ASDAS-CRP | 1.7 (0.9) | 2.1 (1.0) | 0.058 |
| BASDAI | 2.8 (2.1) | 3.9 (2.6) | 0.024 |
| NSAIDs, ongoing, n (%) | 140 (69%) | 14 (36%) | <0.001 |
| bDMARDs, ongoing, n (%) | 82 (40%) | 21 (54%) | 0.114 |
| csDMARDs, ongoing, n (%) | 39 (19%) | 9 (23%) | 0.660 |

All results are presented as mean (SD) unless otherwise indicated. Group comparisons by Student’s t-test, Mann-Whitney U-test, Chi-square test, and Fisher’s exact test, as appropriate. Missing data: symptom duration 1, HLA-B27 status 1, SJC 5, TJC 5, CRP 1, ASDAS-CRP 6, BASDAI 7. ASDAS-CRP, ankylosing spondylitis disease activity score with CRP; AxSpA, axial spondyloarthritis; BASDAI, Bath ankylosing spondylitis disease activity index; bDMARDs, biologic disease-modifying antirheumatic drugs; cDMARDs, conventional synthetic DMARDs; CRP, C-reactive protein; r-axSpA, radiographic axSpA; SJC, swollen joint count; TJC, tender joint count. P-values <0.05 considered significant.

**
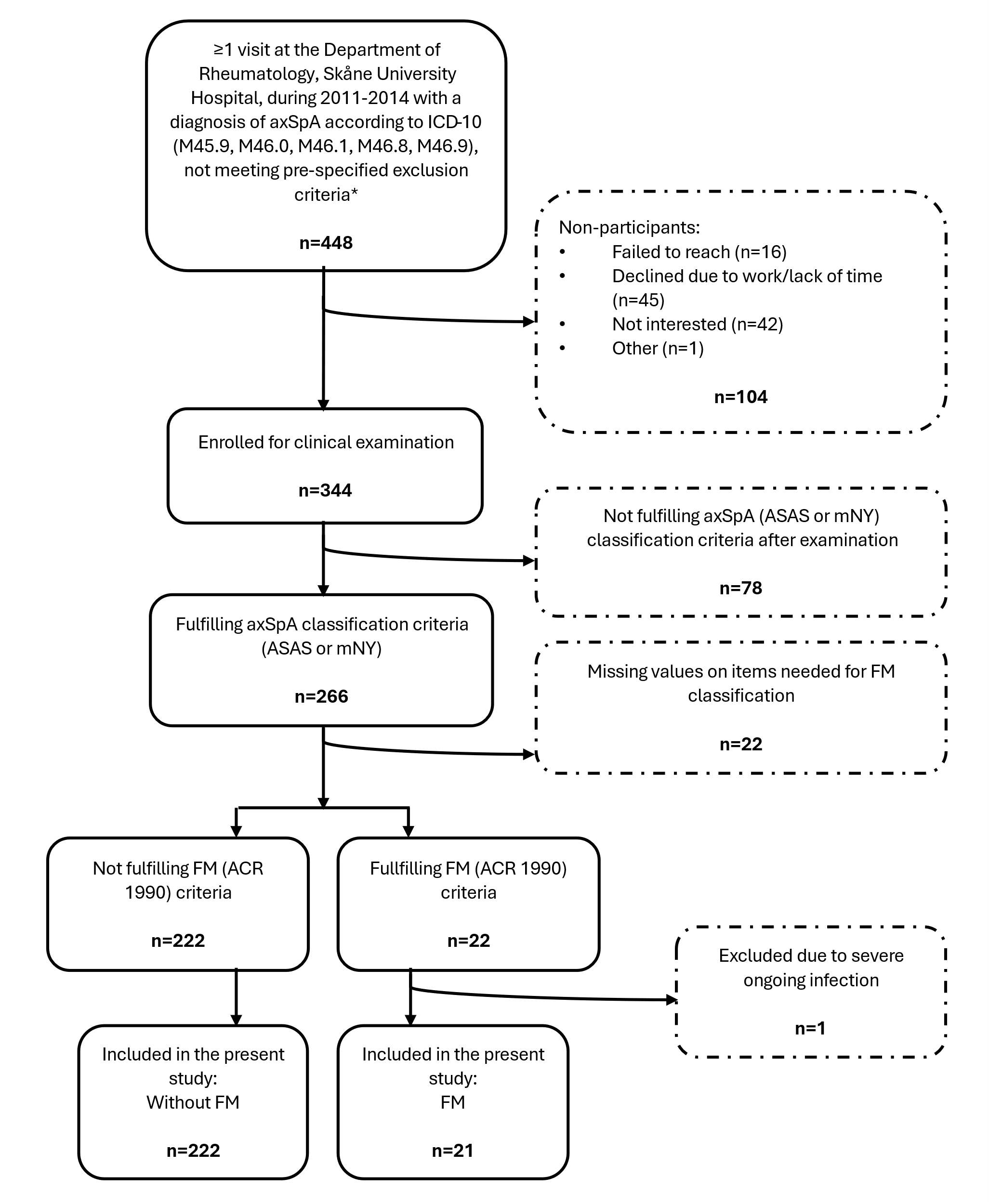
**

**Supplementary Figure S1.** Flowchart of inclusion in the present study regarding comorbid fibromyalgia in axSpA patients from the SPARTAKUS cohort. ASAS, Assessment in SpondyloArthritis international Society; AxSpA, axial spondyloarthritis; FM, fibromyalgia; mNY, modified New York.

*Pre-specified exclusion criteria: deceased, wrong diagnosis code apparent at screening, no back-pain ≥3 months with onset before 45 years of age, other severe medical condition preventing participation, not longer residing in the study region (in Skåne county), not speaking Swedish.

**REFERENCES TO SUPPLEMENTARY MATERIAL**

1. Kvistgaard Olsen J, Fener DK, Waehrens EE, et al. Reliability of Pain Measurements Using Computerized Cuff Algometry: A DoloCuff Reliability and Agreement Study. *Pain Pract*. Jul 2017;17:708-17. doi:10.1111/papr.12514

2. Jespersen A, Amris K, Graven-Nielsen T, et al. Assessment of pressure-pain thresholds and central sensitization of pain in lateral epicondylalgia. *Pain Med*. Feb 2013;14:297-304. doi:10.1111/pme.12021
